# Associating plasma aldosterone concentration with the prevalence of MAFLD in hypertensive patients: insights from a large-scale cross-sectional study

**DOI:** 10.3389/fendo.2024.1451383

**Published:** 2024-09-19

**Authors:** Di Shen, Xintian Cai, Junli Hu, Shuaiwei Song, Qing Zhu, Huimin Ma, Yingying Zhang, Rui Ma, Pan Zhou, Wenbo Yang, Jing Hong, Delian Zhang, Nanfang Li

**Affiliations:** Hypertension Center of People’s Hospital of Xinjiang Uygur Autonomous Region, Xinjiang Hypertension Institute, NHC Key Laboratory of Hypertension Clinical Research, Key Laboratory of Xinjiang Uygur Autonomous Region, Hypertension Research Laboratory, Xinjiang Clinical Medical Research Center for Hypertension (Cardio-Cerebrovascular) Diseases, Urumqi, Xinjiang, China

**Keywords:** plasma aldosterone concentration, metabolic-dysfunction-associated fatty liver disease, hypertension, cross-sectional study, risk factors

## Abstract

**Objective:**

To explore the link between plasma aldosterone concentration (PAC) and the prevalence of metabolic dysfunction-related fatty liver disease (MAFLD) in hypertensive patients.

**Methods:**

We analyzed data from 41,131 hospitalized patients from January 1, 2014, to December 31, 2023. Multivariate logistic regression models tested associations, with threshold, subgroup, and sensitivity analyses conducted to validate findings.

**Results:**

For each 5-unit increase in PAC, the risk of MAFLD rose by 1.57 times, consistent even in the fully adjusted model. The odds ratios for the Q2, Q3, and Q4 groups compared to Q1 were 1.21, 2.12, and 3.14, respectively. A threshold effect was observed at 14 ng/dL, with subgroup and sensitivity analyses supporting these results.

**Conclusions:**

This study reveals a significant positive association between elevated PAC levels and the prevalence of MAFLD in hypertensive patients. These findings underscore the imperative for further large-scale, prospective studies to validate and expand upon this correlation.

## 1 Introduction

Metabolic-dysfunction-associated fatty liver disease (MAFLD), a prevalent condition that affects approximately one-quarter of the global adult population, represents a significant health and economic burden across all societies, with a notable impact on the Asian demographic (1–4). In 2019, a consensus was reached by an international panel of experts who proposed the term “MAFLD” to more accurately encapsulate the condition, regardless of alcohol consumption or the presence of other concurrent liver pathologies. This nomenclature underscores the centrality of metabolic dysfunction in the etiology, clinical presentation, progression, and outcomes of hepatic steatosis (1, 5–7). MAFLD is recognized as the hepatic manifestation of a broader multisystem disorder, characterized by heterogeneity in its etiologies, manifestations, clinical course, and outcomes (8–10). Epidemiological data indicate that the prevalence of MAFLD in Asian countries varies from 10% to 30% and is on an ascending trend (11–15). Hypertension, a chronic condition with a substantial global incidence, is a well-established risk factor for cardiovascular diseases (CVD) (16–18). Moreover, MAFLD has been shown to intensify the progression of atherosclerosis and heighten the risk of cardiovascular events (19, 20). Emerging research has delineated a bidirectional relationship between MAFLD and hypertension, with evidence implicating MAFLD as both a consequence and a precipitant of hypertensive conditions (21). The co-occurrence of hypertension and MAFLD has been associated with more adverse cardiovascular outcomes than either condition in isolation (22). Therefore, early identification, management, and intervention for the combined burden of MAFLD and hypertension are of critical importance, with far-reaching implications for public health (23).

Previous research on MAFLD has primarily focused on factors such as insulin resistance, metabolic syndrome, genetic predisposition, excessive obesity, and lifestyle influences, while notably overlooking the impact of aldosterone (1, 5, 9, 24). Aldosterone, a steroid hormone produced by the adrenal zona glomerulosa, plays a vital role in regulating sodium and water balance in the body, which significantly affects blood pressure control (25–31). Numerous studies have identified the excessive secretion of aldosterone as a major risk factor for cardiovascular and kidney diseases, as well as metabolic disorders (26, 28, 29, 32, 33). Furthermore, recent research suggests that plasma aldosterone concentration (PAC) can influence liver metabolism (34, 35). For instance, studies have revealed a nonlinear relationship between elevated PAC levels and the incidence of non-alcoholic fatty liver disease (NAFLD) in patients with hypertension (36). Additionally, in animal models, aldosterone receptor blockers have been found to inhibit hepatic stellate cells and reduce liver fibrosis, indicating their potential effectiveness in treating fatty liver disease (37).

However, the relationship between PAC and MAFLD, a newer term for metabolic fatty liver disease, remains unexplored and unclear. This study aims to delineate the correlation between PAC and MAFLD, aspiring to provide innovative insights into the prevention and therapeutic management of metabolic fat deposition, particularly within the hypertensive patient population.

## 2 Material and methods

### 2.1 Study population

**Figure 1:**
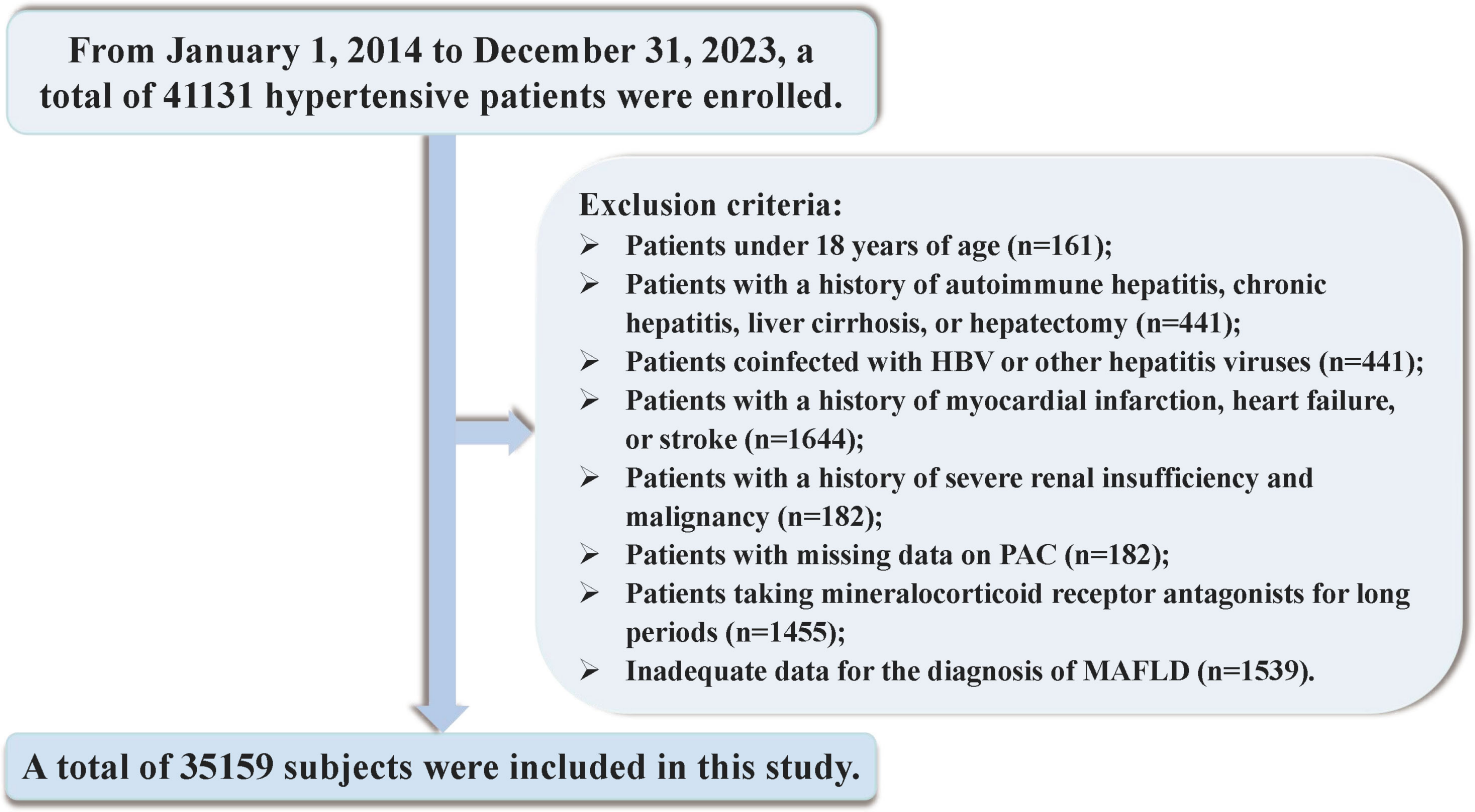
Flowchart of the study.

Between January 1, 2014, and December 31, 2023, a total of 41131 hospitalized patients were included in the study. Exclusion criteria were applied to participants under the age of 18 and those with incomplete data on PAC or insufficient information for the diagnosis of MAFLD. To mitigate the potential confounding effects of certain conditions and medications on study outcomes, we meticulously excluded individuals with positive serology for hepatitis B, C, or Delta viruses, autoimmune hepatitis, cirrhosis, history of liver resection, liver cancer, or gastrointestinal surgery. Furthermore, participants with a diagnosis of endocrine hypertension, severe thyroid disorders, chronic use of mineralocorticoid receptor antagonists, recent severe cardiovascular or cerebrovascular events, significant hepatic or renal impairment, or malignancies diagnosed within the preceding three months were also excluded. Individuals with a history of heavy alcohol consumption were additionally excluded to account for the impact on liver metabolism. After these exclusions, 35159 participants were included in the final analysis (**Figure 1**). Informed consent was obtained in writing from all patients or their legal guardians, and the study was approved by the hospital’s ethics committee. Adherence to the STROBE guidelines was ensured in the reporting of this research (38).

### 2.2 Data collection and definitions

Data including clinical information, test findings, lifestyle variables, medical history, and medication history were obtained from the electronic medical record as baseline. Clinical data at admission included age, sex, height, weight, body mass index (BMI), systolic, diastolic, and waist circumference (WC). Smoking and alcohol drinking were classified as current or non-current. For specific measurement methods, please refer to the **Supplementary Material**.

Peripheral venous blood was collected after an 8–10 hour fast to measure serum potassium, serum sodium, platelets (PLT), alanine aminotransferase (ALT), aspartate aminotransferase (AST), gammaglutamyl transferase (GGT), serum creatinine (Scr), uric acid(UA), total cholesterol (TC), triglycerides (TG), high-density lipoprotein cholesterol (HDL-C), low-density lipoprotein cholesterol(LDL-C), HbA1c,high sensitivity C-reactive protein (hs-CRP), andtriglyceride–glucose (TyG) index. The above blood biochemical indicators were detected by Automatic Analyzer (7600-010, Hitachi, Tokyo, Japan) according to the manufactures instruction. Thehexokinase/glucose-6-phosphate dehydrogenase method was used to measure FBG levels, while TG levels were measured using the enzymatic colorimetric method. Before calculation, the units of TG and FBG were converted from mmol/L to mg/dL (For TG, 1 mmol/l = 88.57 mg/dl; For FBG, 1 mmol/l = 18 mg/dL), and the TyG index was then calculated as ln [TG (mg/dL) × FBG (mg/dL)/2]. Hormone measurements are based on current guidelines and our previous studies. The PAC was measured by radioimmunoassay (DSL-8600; DSL, Webster, TX) (29, 36, 39). Please refer to the **Supplementary Material** for detailed definitions of the diseases.

### 2.3 Outcome

Hepatic steatosis with evidence of metabolic dysfunction defines MAFLD (11). Metabolic dysfunction was defined as satisfying one of the following three conditions: overweight or obese (BMI ≥ 23 kg/m^2^); type 2 diabetes mellitus (T2DM); metabolic abnormality score ≥ 2 (WC ≥ 90 cm in men and ≥ 80 cm in women; blood pressure ≥ 130/85 mmHg or use of antihypertensives; TG ≥ 150 mg/dL or use of antidyslipidemics; HDL-C < 40 mg/dL in men and < 50 mg/dL in women or use of antidyslipidemics; FBG 5.6-6.9 mmol/L; hs-CRP > 2 mg/L; homeostasis model assessment of insulin resistance score [HOMA-IR] ≥ 2.5). For lack of information on HOMA-IR, we used the TyG index over the 75th percentile as an alternative to the HOMA-IR diagnostic criteria (40, 41).

### 2.4 Statistical analysis

Multicollinearity was assessed using the variance inflation factortest (**Supplementary Table S1**). The relationship between PAC levels and MAFLD was analyzed using a multivariate logistic regression model, and the odds ratio (OR) was calculated. Additionally, we used restricted cubic splines (RCS) to evaluate the dose-response relationship and conducted a two-stage comparative analysis based on the inflection points of the RCS curve. Additionally, subgroup analyses were conducted to ascertain the influence of PAC on MAFLD across a spectrum of stratifying variables. Several extra sensitivity analyses were performed to assess the reliability of the results. **Supplementary Materials** provide details on the statistical analysis.

The statistical analysis was executed using R software, version 4.1.1. Significance was defined as a p-value of less than 0.05, employing a two-tailed test for statistical inference.

## 3 Results

### 3.1 Baseline characteristics

**Figure 2:**
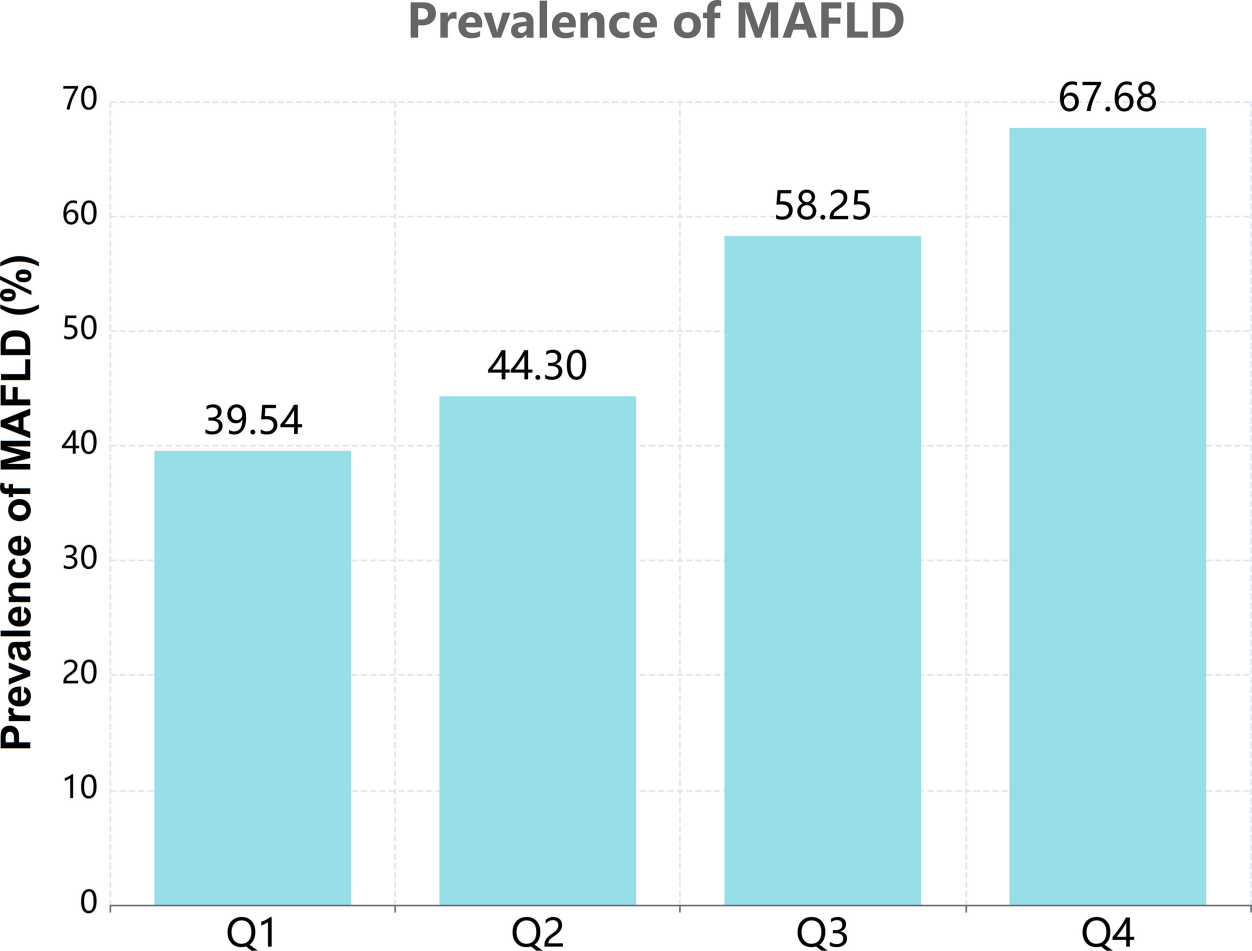
The prevalence of MAFLD according to the quartiles of PAC.

Based on the quartiles of PAC, baseline characteristics of each group are presented in **Table 1**. A total of 35159 patients were included, among which 20078 (57.11%) were male. The high PAC group was younger, comprised more females, and had a higher BMI. In terms of test indicators, Scr, WC, and TyG indexes were significantly higher in the high PAC group compared to the low PAC group. Moreover, the high PAC group were more likely to be taking diuretics and calcium channel blockers (CCB) (**Table 1**). The most significant discovery was that the prevalence of MAFLD appeared to rise as PAC increased (**Figure 2**). Furthermore, after dichotomizing according to the presence or absence of MAFLD, there were significant differences in PAC, age, TyG index, and smoking history between the two groups (**Supplementary Table S2**).

**Table 1:** Baseline characteristics according to quartiles of PAC.

Variables	Q1	Q2	Q3	Q4	P-value
	(<11.83ng/dL)	(11.83-14.08ng/dL)	(14.08-18.65ng/dL)	(>18.65ng/dL)	
Sample size, n	8790	8790	8790	8789	
Demography					
Age, years	52.05 ± 12.58	51.40 ± 11.48	50.54 ± 12.07	49.50 ± 12.03	<0.001*
Sex, %					0.037*
Women	3705 (42.15%)	3790 (43.12%)	3714 (42.25%)	3872 (44.06%)	
Men	5085 (57.85%)	5000 (56.88%)	5076 (57.75%)	4917 (55.94%)	
BMI, kg/m^2^	26.86 ± 3.68	26.93 ± 3.66	26.95 ± 3.61	26.98 ± 3.60	0.163
WC, cm	95.63 ± 11.34	95.84 ± 11.25	95.91 ± 11.19	96.02 ± 11.13	0.128
SBP, mmHg	146.13 ± 18.39	146.07 ± 18.20	145.70 ± 18.24	146.17 ± 18.34	0.31
DBP, mmHg	88.34 ± 13.67	88.14 ± 13.56	87.82 ± 13.48	88.14 ± 13.67	0.094
Current smoking, %	3115 (35.44%)	2991 (34.03%)	2959 (33.66%)	2647 (30.12%)	<0.001*
Current drinking, %	2769 (31.50%)	2762 (31.42%)	2764 (31.44%)	2553 (29.05%)	<0.001*
Biochemical indexes					
Serum potassium (mmol/L)	4.07 ± 0.29	3.98 ± 0.27	3.83 ± 0.31	3.63 ± 0.28	<0.001*
Serum sodium (mmol/L)	141.11 ± 2.55	141.11 ± 2.45	141.03 ± 2.49	141.04 ± 2.58	0.089
PLT, 10^9/L	242.29 ± 58.68	243.15 ± 58.15	242.90 ± 57.83	240.01 ± 57.66	0.001*
ALT, U/L	27.36 ± 17.54	27.17 ± 17.54	27.36 ± 17.60	27.25 ± 17.42	0.871
AST, U/L	21.10 ± 8.24	21.00 ± 8.16	21.08 ± 8.17	21.05 ± 8.31	0.877
GGT, U/L	36.25 ± 25.77	35.53 ± 24.80	35.86 ± 25.17	36.09 ± 25.30	0.261
Scr, µmol/L	64.93 ± 14.46	65.03 ± 14.32	65.05 ± 14.37	65.43 ± 14.48	0.101
BUN, mmol/L	5.05 ± 1.36	5.04 ± 1.35	5.05 ± 1.36	5.08 ± 1.36	0.325
UA, umol/L	343.61 ± 91.51	342.97 ± 90.57	343.16 ± 90.80	345.74 ± 91.23	0.161
Total cholesterol, mmol/L	4.55 ± 0.99	4.51 ± 0.97	4.55 ± 0.98	4.53 ± 0.97	0.032*
Triglyceride, mmol/L	1.82 ± 1.04	1.80 ± 1.01	1.81 ± 1.03	1.82 ± 1.06	0.439
HDL-C, mmol/L	1.06 ± 0.25	1.05 ± 0.24	1.06 ± 0.25	1.06 ± 0.25	0.039*
LDL-C, mmol/L	2.73 ± 0.83	2.74 ± 0.82	2.78 ± 0.82	2.77 ± 0.82	<0.001*
FBG, mmol/L	5.05 ± 1.07	5.03 ± 1.04	5.00 ± 1.02	5.03 ± 1.05	0.032*
HbA1c, %	5.99 ± 0.82	5.95 ± 0.79	5.91 ± 0.75	5.89 ± 0.79	<0.001*
hs-CRP, mg/dL	3.40 ± 3.01	3.58 ± 3.12	3.50 ± 3.03	3.54 ± 3.13	<0.001*
TyG index	6.53 ± 0.52	6.89 ± 0.11	7.27 ± 0.12	7.98 ± 0.47	<0.001*
Previous history					
T2DM, %	1524 (17.34%)	1384 (15.75%)	1324 (15.06%)	1406 (16.00%)	<0.001*
Dyslipidemia, %	1676 (19.07%)	1682 (19.14%)	1767 (20.10%)	1540 (17.52%)	<0.001*
CAD, %	944 (10.74%)	808 (9.19%)	746 (8.49%)	745 (8.48%)	<0.001*
Medications use					
ACEI/ARB, %	4235 (48.18%)	4016 (45.69%)	3942 (44.85%)	4034 (45.90%)	<0.001*
Diuretic, %	905 (10.30%)	893 (10.16%)	937 (10.66%)	1052 (11.97%)	<0.001*
CCB, %	2021 (22.99%)	2069 (23.54%)	2228 (25.35%)	2665 (30.32%)	<0.001*
β-blockers, %	1688 (19.20%)	1542 (17.54%)	1489 (16.94%)	1505 (17.12%)	<0.001*
Antidiabetic agents, %	758 (8.62%)	676 (7.69%)	570 (6.48%)	612 (6.96%)	<0.001*
Lipid-lowering drugs, %	1139 (12.96%)	1050 (11.95%)	980 (11.15%)	928 (10.56%)	<0.001*

Data are mean (standard deviation), n (%), or median (interquartile range).

BMI, body mass index; SBP, systolic blood pressure; DBP, diastolic blood pressure; WC, waist circumference; PLT, platelets; ALT, alanine aminotransferase; AST, aspartate aminotransferase; GGT, gamma glutamyl transferase; Scr, serumcreatinine; BUN, blood urea nitrogen; UA, uric acid; HDL-C, high-density lipoprotein cholesterol; LDL-C, low-density lipoprotein cholesterol; FBG, fasing blood glucose; HbA1c, glycosylated hemoglobin; hs-CRP, high sensitivity C-reactive protein; TyG index, triglyceride glucose index; DM, diabetes mellitus; CAD, coronary artery disease; ACEI, angiotensin-converting enzyme inhibitor; ARB, angiotensin receptor blocker; CCB, calcium channel blockers.

*Significant P value (P < 0.05).

### 3.2 Relationship between PAC and the prevalence of MAFLD

**Figure 3:**
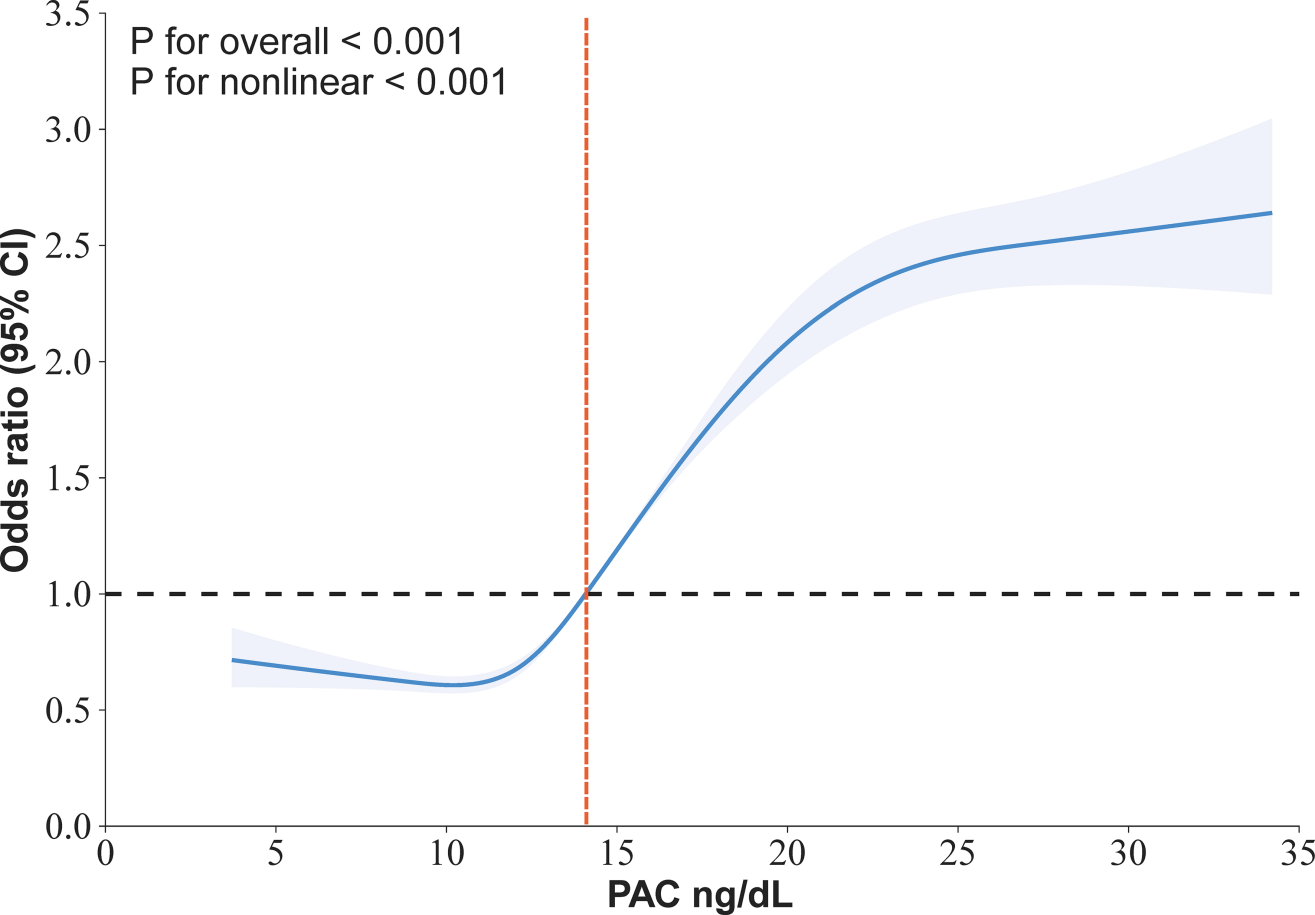
Restricted cubic spline for the association between PAC and the prevalence of MAFLD in in hypertensive patients.

Our research has found a close association between PAC and MAFLD. In the original model, for every 5-unit increase in PAC, the risk of developing MAFLD increases by 1.57 times. This relationship remains reliable in the fully adjusted model. Compared to the Q1 group, the OR values for the Q2, Q3, and Q4 groups are 1.21, 2.12, and 3.14 respectively, showing an increasing trend (**Table 2**). We further utilized the RCS model to identify the nonlinear dose-response relationship between PAC levels and the prevalence of MAFLD (*p* for nonlinear < 0.001) (**Figure 3**). Furthermore, we conducted a two-stage comparative analysis based on the inflection point of RCS. The results indicate that the risk of developing MAFLD for individuals with a PAC level greater than 14ng/dL is 2.32 times higher than that of individuals with a level less than 14ng/dL (**Table 3**).

**Table 2:** The relationship between PAC and the prevalence of MAFLD in hypertensive patients.

Exposure	Model 1 OR (95% CI)	Model 2 OR (95% CI)	Model 3 OR (95% CI)	Model 4 OR (95% CI)	Model 5 OR (95% CI)
PAC (per 5 ng/dl increase)	1.57 (1.54, 1.61)	1.57 (1.54, 1.60)	1.57 (1.54, 1.60)	1.56 (1.53, 1.59)	1.56 (1.53, 1.60)
PAC quartiles					
Q1 (<11.83)	Reference	Reference	Reference	Reference	Reference
Q2 (11.83-14.08)	1.21 (1.14, 1.29)	1.21 (1.14, 1.29)	1.21 (1.14, 1.29)	1.21 (1.14, 1.28)	1.21 (1.14, 1.29)
Q3 (14.08-18.65)	2.13 (2.00, 2.26)	2.13 (2.00, 2.26)	2.13 (2.00, 2.26)	2.11 (1.99, 2.25)	2.12 (1.99, 2.25)
Q4 (>18.65)	3.20 (3.01, 3.40)	3.19 (3.00, 3.39)	3.19 (2.99, 3.39)	3.12 (2.93, 3.32)	3.14 (2.95, 3.35)
P for trend	<0.001	<0.001	<0.001	<0.001	<0.001

Model 1: crude model.

Model 2: adjusted for age, sex, smoking status, alcohol consumption, BMI, SBP, and DBP.

Model 3: adjusted for variables in Model 2 plus DM, dyslipidemia, CAD.

Model 4: adjusted for variables in Model 3 plus Serum potassium, Serum sodium, PLT, ALT, AST, GGT, Scr, BUN, UA, Total cholesterol, Triglyceride, HDL-C, LDL-C, FBG, HbA1c, hs-CRP, TyG index.

Model 5: adjusted for variables in Model 4 plus ACEI/ARB, diuretic, CCB, β-blockers, antidiabetic agents, lipid-lowering drugs.

SD, standard deviation; OR, Odds ratio. Other abbreviations, see **Table 1**.

**Table 3:** Analysis of the prevalence of MAFLD Based on RCS Turning Point.

Exposure	Model 1 OR (95% CI),	Model 2 OR (95% CI)	Model 3 OR (95% CI)	Model 4 OR (95% CI)	Model 5 OR (95% CI)
PAC					
<14 ng/dL	Reference	Reference	Reference	Reference	Reference
≥ 14 ng/dL	2.35 (2.25, 2.45)	2.34 (2.24, 2.44)	2.34 (2.24, 2.44)	2.31 (2.22, 2.41)	2.32 (2.22, 2.42)

Model 1: crude model.

Model 2: adjusted for age, sex, smoking status, alcohol consumption, BMI, SBP, and DBP.

Model 3: adjusted for variables in Model 2 plus DM, dyslipidemia, CAD.

Model 4: adjusted for variables in Model 3 plus Serum potassium, Serum sodium, PLT, ALT, AST, GGT, Scr, BUN, UA, Total cholesterol, Triglyceride, HDL-C, LDL-C, FBG, HbA1c, hs-CRP, TyG index.

Model 5: adjusted for variables in Model 4 plus ACEI/ARB, diuretic, CCB, β-blockers, antidiabetic agents, lipid-lowering drugs.

SD, standard deviation; OR, odds ratio; CI, confidence interval. Other abbreviations, see **Table 1**.

### 3.3 Subgroup analysis

**Figure 4:**
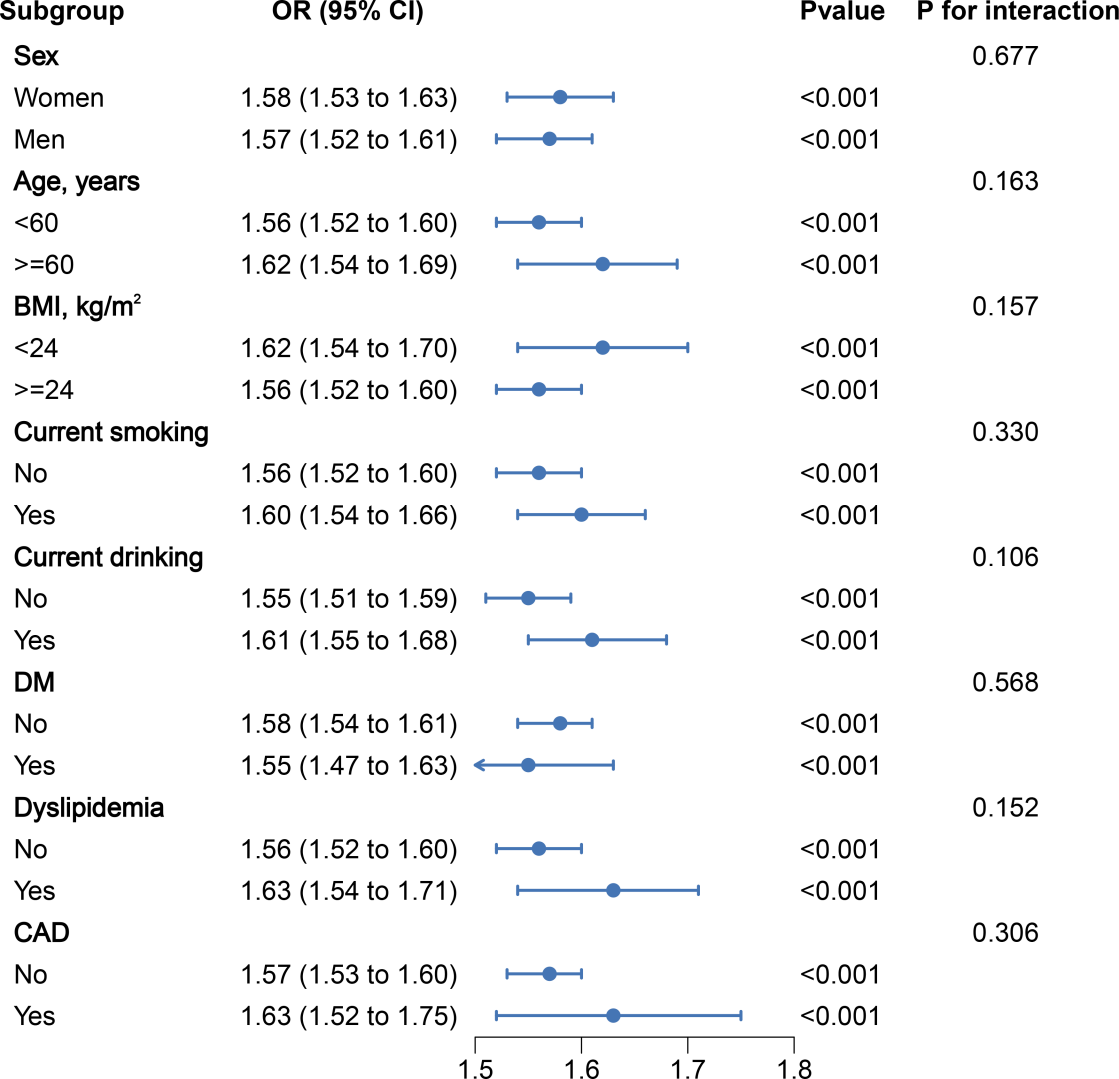
Stratified analyses of the association between PAC (per 5 ng/dL increment) and the prevalence of MAFLDin hypertensive patients.

After stratifying the data based on basic conditions and diseases, the results remained stable across the population, with no interactions observed (**Figure 4**). In addition, a subgroup analysis examined the potential impact of antihypertensive drugs on outcomes, which also showed no significant changes (**Supplementary Figure S1**). This indicates that our findings are not affected by these stratification factors and that PAC can predict the occurrence of MAFLD regardless of stratification. In order to eliminate any bias caused by missing data, we conducted sensitivity analyses after excluding patients with missing values and obtained essentially the same results (**Supplementary Table S3**). Furthermore, to mitigate the impact of alcohol abuse on the results, we excluded patients with a history of alcohol abuse and the results remained consistent (**Supplementary Table S4**). Additionally, to eliminate the influence of severe liver fibrosis on aldosterone inactivation, we excluded patients with severe liver fibrosis. The correlation between PAC and the prevalence of MAFLD remained largely unchanged (**Supplementary Tables S5, S6**). Additionally, to assess the influence of unmeasured confounders, we conducted an E-value analysis, which indicated that the impact of confounding factors was minimal and the likelihood of our results being overturned was low (**Supplementary Table S7** and **Supplementary Figure S2**).

## 4 Discussion

Our study has, for the first time, uncovered the relationship between PAC levels and the prevalence of MAFLD in hypertensive patients, revealing an independent association between elevated PAC and an increased incidence of MAFLD. Our findings indicate that individuals with PAC levels exceeding 14 ng/dL exhibit a 2.32-fold heightened risk for the development of MAFLD compared to those with levels below this threshold. This suggests that maintaining PAC within a reasonable range may offer a new direction for preventing MAFLD in the future.

Aldosterone, a crucial mineralocorticoid hormone, plays an essential role in regulating the body’s water and electrolyte balance (42–45). While its excess is known to indicate hypertension and organ damage, its connection to liver steatosis remains underexplored. Fallo and colleagues highlight that patients with primary aldosteronism are more prone to insulin resistance and have a higher prevalence of NAFLD, suggesting an increased risk of metabolic and liver diseases in this subgroup (46). Additionally, a large cohort study observed that angiotensin-converting enzyme inhibitors are linked to a reduced risk of adverse liver events in liver steatosis patients (47). Spironolactone, an aldosterone receptor antagonist, has shown beneficial effects on serum insulin and HOMA-IR in NAFLD patients, according to animal studies (48). Further supporting this, the Jackson Heart Study, involving 2,507 participants, found a positive correlation between aldosterone levels and fatty liver in African American women (49). Research by Srinivasa et al. indicates that elevated aldosterone could be a risk factor for liver fat accumulation in HIV-infected individuals (50). A recent study shows that in hypertensive patients, the risk of developing new-onset NAFLD significantly increases when PAC levels are ≥13 ng/dL (36). This research overcomes previous studies’ limitations, such as animal reliance, limited populations, small samples, and inadequate variable adjustments, providing a more comprehensive analysis.

The specific mechanisms by which PAC leads to the development of MAFLD remain unclear and may involve several potential pathways. First, an excess of aldosterone can enhance oxidative stress and inflammation, which could potentially lead to liver damage and the progression of fatty liver disease (34, 51–54). Second, an overabundance of aldosterone can also cause a decrease in adiponectin levels in the bloodstream, and a corresponding reduction in its expression in visceral adipose tissue. This hormone, known as adiponectin, plays a crucial regulatory role in fat storage and reducing insulin resistance (30, 55–57). Third, aldosterone can trigger a direct sequence of events leading to the activation of hepatic stellate cells (HSC) and eventually liver fibrosis, primarily by inducing the activation of the NLRP3 inflammasome (58). Finally, more recent research has shed light on the fact that aldosterone can be locally produced during the process of liver fibrinogenesis, thereby contributing to organ fibrosis (31, 35, 59). To counter these effects, the therapeutic efficacy of aldosterone antagonists has been recognized. Existing studies have reliably shown that certain aldosterone antagonists, notably spironolactone and eplerenone, can diminish the symptoms of fatty liver and liver fibrosis (33, 58, 60, 61). This significantly underlines the critical role that aldosterone plays in the pathophysiology of fatty liver disease.

Our study’s strengths lie in the large sample size, strict exclusion criteria, and the first-time revelation of the relationship between PAC and MAFLD. We utilized multiple statistical methods to further validate the reliability of our research findings. These groundbreaking results may also offer new insights into the early identification and intervention of MAFLD in hypertensive patients. However, while considering these advantages, we must also acknowledge that our study may have some limitations. Firstly, our study is cross-sectional in design, which prevents us from establishing a causal relationship between PAC and MAFLD. Secondly, we did not take into account the potential influence of confounding factors such as dietary habits and level of physical activity, so we conducted an E-value analysis. The results indicate that the likelihood of our findings being overturned is very low. Thirdly, rather than using liver biopsy, abdominal ultrasound was used to diagnose fatty liver. Ultrasound, however, has good accuracy in non-invasive detection of fatty liver, making it widely used in clinical practice and epidemiological studies. Fourthly, our study was limited to hypertensive patients in China, and the generalizability of the conclusions may be affected, necessitating further research in a more diverse and broader patient population to validate our findings.

## 5 Conclusion

This study revealed a groundbreaking positive relationship between PAC and the prevalence of MAFLD, particularly with a significant increase in the risk of developing MAFLD when PAC exceeds 14ng/dL. This further suggests that maintaining PAC at a reasonable level may be beneficial in preventing the occurrence of MAFLD in hypertensive patients. However, to validate and confirm these findings, it is necessary to conduct more large-scale prospective studies in the future.

## Data Availability

The original contributions presented in the study are included in the article/**Supplementary Material**. Further inquiries can be directed to the corresponding author.

## References

[1] Eslam M Sanyal AJ George J International Consensus Panel. MAFLD: A consensus-driven proposed nomenclature for metabolic associated fatty liver disease. Gastroenterology. (2020) 158:1999–2014.e1. doi: 10.1053/j.gastro.2019.11.312 32044314

[2] Younossi Z Anstee QM Marietti M Hardy T Henry L Eslam M. Global burden of NAFLD and NASH: trends, predictions, risk factors and prevention. Nat Rev Gastroenterol Hepatol. (2018) 15:11–20. doi: 10.1038/nrgastro.2017.109 28930295

[3] Sarin SK Kumar M Eslam M George J Al Mahtab M Akbar S. Liver diseases in the Asia-Pacific region: a Lancet Gastroenterology & Hepatology Commission. Lancet Gastroenterol Hepatol. (2020) 5:167–228. doi: 10.1016/S2468-1253(19)30342-5 31852635 PMC7164809

[4] MaChado MV Cortez-Pinto H. NAFLD, MAFLD and obesity: brothers in arms. Nat Rev Gastroenterol Hepatol. (2023) 20:67–8. doi: 10.1038/s41575-022-00717-4 36470966

[5] Vitale A Svegliati-Baroni G Ortolani A Cucco M Dalla Riva GV Giannini EG. Epidemiological trends and trajectories of MAFLD-associated hepatocellular carcinoma 2002-2033: the ITA.LI.CA database. Gut. (2023) 72:141–52. doi: 10.1136/gutjnl-2021-324915 34933916

[6] Nan Y An J Bao J Chen H Chen Y Ding H. The Chinese Society of Hepatology position statement on the redefinition of fatty liver disease. J Hepatol. (2021) 75:454–61. doi: 10.1016/j.jhep.2021.05.003 34019941

[7] Zhang XL Fan JG Wei L Shi JP Zheng MH. Promoting the term MAFLD: China in action. Lancet Gastroenterol Hepatol. (2022) 7:598. doi: 10.1016/S2468-1253(22)00127-3 35709820

[8] Byrne CD Targher G. NAFLD: a multisystem disease. J Hepatol. (2015) 62:S47–64. doi: 10.1016/j.jhep.2014.12.012 25920090

[9] Guo B Guo Y Nima Q Feng Y Wang Z Lu R. Exposure to air pollution is associated with an increased risk of metabolic dysfunction-associated fatty liver disease. J Hepatol. (2022) 76:518–25. doi: 10.1016/j.jhep.2021.10.016 34883157

[10] Kim D Konyn P Sandhu KK Dennis BB Cheung AC Ahmed A. Metabolic dysfunction-associated fatty liver disease is associated with increased all-cause mortality in the United States. J Hepatol. (2021) 75:1284–91. doi: 10.1016/j.jhep.2021.07.035 34380057

[11] Eslam M Newsome PN Sarin SK Anstee QM Targher G Romero-Gomez M. A new definition for metabolic dysfunction-associated fatty liver disease: An international expert consensus statement. J Hepatol. (2020) 73:202–9. doi: 10.1016/j.jhep.2020.03.039 32278004

[12] Zheng KI Sun DQ Jin Y Zhu PW Zheng MH. Clinical utility of the MAFLD definition. J Hepatol. (2021) 74:989–91. doi: 10.1016/j.jhep.2020.12.016 33347953

[13] Zhou XD Lonardo A Pan CQ Shapiro MD Zheng MH. Clinical features and long-term outcomes of patients diagnosed with MASLD, MAFLD, or both. J Hepatol. (2024), S0168-8278(24)00223-00223X [pii]. doi: 10.1016/j.jhep.2024.03.039 38554846

[14] Cai X Wang M Liu S Yuan Y Hu J Zhu Q. Establishment and validation of a nomogram that predicts the risk of type 2 diabetes in obese patients with non-alcoholic fatty liver disease: a longitudinal observational study. Am J Transl Res. (2022) 14:4505–14. PMC9360847 35958467

[15] Cai X Aierken X Ahmat A Cao Y Zhu Q Wu T. A nomogram model based on noninvasive bioindicators to predict 3-year risk of nonalcoholic fatty liver in nonobese mainland chinese: A prospective cohort study. BioMed Res Int. (2020) 2020:8852198. doi: 10.1155/2020/8852198 33204721 PMC7655259

[16] Zhang M Shi Y Zhou B Huang Z Zhao Z Li C. Prevalence, awareness, treatment, and control of hypertension in China, 2004-18: findings from six rounds of a national survey. BMJ. (2023) 380:e071952. doi: 10.1136/bmj-2022-071952 36631148 PMC10498511

[17] Krist AH Davidson KW Mangione CM Cabana M Caughey AB Davis EM. Screening for hypertension in adults: US preventive services task force reaffirmation recommendation statement. JAMA. (2021) 325:1650–6. doi: 10.1001/jama.2021.4987 33904861

[18] Mancia G Fagard R Narkiewicz K Redón J Zanchetti A Böhm M. 2013 ESH/ESC Guidelines for the management of arterial hypertension: the Task Force for the management of arterial hypertension of the European Society of Hypertension (ESH) and of the European Society of Cardiology (ESC). J Hypertens. (2013) 31:1281–357. doi: 10.1097/01.hjh.0000431740.32696.cc 23817082

[19] Targher G Byrne CD Tilg H. NAFLD and increased risk of cardiovascular disease: clinical associations, pathophysiological mechanisms and pharmacological implications. Gut. (2020) 69:1691–705. doi: 10.1136/gutjnl-2020-320622 32321858

[20] GutiGuti.ns.alca J Santos A Armendariz-Borunda J. Pathophysiological molecular mechanisms of obesity: A link between MAFLD and NASH with cardiovascular diseases. Int J Mol Sci. (2021) 22:11629. doi: 10.3390/ijms222111629 34769060 PMC8583943

[21] Ma J Hwang SJ Pedley A Massaro JM Hoffmann U Chung RT. Bi-directional analysis between fatty liver and cardiovascular disease risk factors. J Hepatol. (2017) 66:390–7. doi: 10.1016/j.jhep.2016.09.022 PMC5250546 27729222

[22] Johnston MP Patel J Byrne CD. Causes of mortality in non-alcoholic fatty liver disease (NAFLD) and alcohol related fatty liver disease (AFLD). Curr Pharm Des. (2020) 26:1079–92. doi: 10.2174/1381612826666200128094231 32003662

[23] Lonardo A Nascimbeni F Mantovani A Targher G. Hypertension, diabetes, atherosclerosis and NASH: Cause or consequence. J Hepatol. (2018) 68:335–52. doi: 10.1016/j.jhep.2017.09.021 29122390

[24] Tong L Chen Z Li Y Wang X Yang C Li Y. Transketolase promotes MAFLD by limiting inosine-induced mitochondrial activity. Cell Metab. (2024) 36:1013–1029.e5. doi: 10.1016/j.cmet.2024.03.003 38547864

[25] Struthers AD MacDonald TM. Review of aldosterone- and angiotensin II-induced target organ damage and prevention. Cardiovasc Res. (2004) 61:663–70. doi: 10.1016/j.cardiores.2003.11.037 14985063

[26] Leopold JA Ingelfinger JR. Aldosterone and treatment-resistant hypertension. N Engl J Med. (2023) 388:464–7. doi: 10.1056/NEJMe2213559 36724334

[27] Williams B. A new dawn for aldosterone as a therapeutic target in hypertension. JAMA. (2023) 330:1138–9. doi: 10.1001/jama.2023.17087 37690088

[28] Tuttle KR Hauske SJ Canziani ME Caramori ML Cherney D Cronin L. Efficacy and safety of aldosterone synthase inhibition with and without empagliflozin for chronic kidney disease: a randomised, controlled, phase 2 trial. Lancet. (2024) 403:379–90. doi: 10.1016/S0140-6736(23)02408-X 38109916

[29] Song S Cai X Hu J Zhu Q Shen D Heizhati M. Plasma aldosterone concentrations elevation in hypertensive patients: the dual impact on hyperuricemia and gout. Front Endocrinol (Lausanne). (2024) 15:1424207. doi: 10.3389/fendo.2024.1424207 39140032 PMC11319118

[30] Joseph JJ Pohlman NK Zhao S Kline D Brock G Echouffo-Tcheugui JB. Association of serum aldosterone and plasma renin activity with ambulatory blood pressure in african americans: the jackson heart study. Circulation. (2021) 143:2355–66. doi: 10.1161/CIRCULATIONAHA.120.050896 PMC8789344 33605160

[31] Verma A Vaidya A Subudhi S Waikar SS. Aldosterone in chronic kidney disease and renal outcomes. Eur Heart J. (2022) 43:3781–91. doi: 10.1093/eurheartj/ehac352 PMC10147385 36219773

[32] Roper RJ Goodlett CR. A new Down syndrome rat model races forward. Trends Genet. (2022) 38:1101–2. doi: 10.1016/j.tig.2022.05.001 PMC9561036 35581033

[33] Laffin LJ Rodman D Luther JM Vaidya A Weir MR Rajicic N. Aldosterone synthase inhibition with lorundrostat for uncontrolled hypertension: the target-HTN randomized clinical trial. JAMA. (2023) 330:1140–50. doi: 10.1001/jama.2023.16029 PMC10493865 37690061

[34] Ackermann D Mordasini D Cheval L Imbert-Teboul M Vogt B Doucet A. Sodium retention and ascites formation in a cholestatic mice model: role of aldosterone and mineralocorticoid receptor. Hepatology. (2007) 46:173–9. doi: 10.1002/(ISSN)1527-3350 17596887

[35] AlQudah M Hale TM Czubryt MP. Targeting the renin-angiotensin-aldosterone system in fibrosis. Matrix Biol. (2020) 91-92:92–108. doi: 10.1016/j.matbio.2020.04.005 32422329 PMC7434656

[36] Hu J Cai X Zhu Q Heizhati M Wen W Luo Q. Relationship between plasma aldosterone concentrations and non-alcoholic fatty liver disease diagnosis in patients with hypertension: A retrospective cohort study. Diabetes Metab Syndr Obes. (2023) 16:1625–36. doi: 10.2147/DMSO.S408722 PMC10257476 37304667

[37] Hirose A Ono M Saibara T Nozaki Y Masuda K Yoshioka A. Angiotensin II type 1 receptor blocker inhibits fibrosis in rat nonalcoholic steatohepatitis. Hepatology. (2007) 45:1375–81. doi: 10.1002/hep.21638 17518368

[38] von Elm E Altman DG Egger M Pocock SJ Gøtzsche PC Vandenbroucke JP. Strengthening the Reporting of Observational Studies in Epidemiology (STROBE) statement: guidelines for reporting observational studies. BMJ. (2007) 335:806–8. doi: 10.1136/bmj.39335.541782.AD PMC2034723 17947786

[39] Cai X Song S Hu J Zhu Q Shen D Yang W. Association of the trajectory of plasma aldosterone concentration with the risk of cardiovascular disease in patients with hypertension: a cohort study. Sci Rep. (2024) 14:4906. doi: 10.1038/s41598-024-54971-4 38418472 PMC10902285

[40] Li Y Wu S Gao J Zhang Y Zuo Y Tian X. Association of stroke with metabolic dysfunction-associated fatty liver disease with and without CKD. Am J Kidney Dis. (2024) 83:477–88. doi: 10.1053/j.ajkd.2023.08.016 37838141

[41] Guerrero-Romero F Simental-Mendía LE González-Ortiz M Martínez-Abundis E Ramos-Zavala MG Hernández-González SO. The product of triglycerides and glucose, a simple measure of insulin sensitivity. Comparison with the euglycemic-hyperinsulinemic clamp. J Clin Endocrinol Metab. (2010) 95:3347–51. doi: 10.1210/jc.2010-0288 20484475

[42] Buglioni A Cannone V Sangaralingham SJ Heublein DM Scott CG Bailey KR. Aldosterone predicts cardiovascular, renal, and metabolic disease in the general community: A 4-year follow-up. J Am Heart Assoc. (2015) 4:e002505. doi: 10.1161/JAHA.115.002505 26702078 PMC4845260

[43] Nagase M Yoshida S Shibata S Nagase T Gotoda T Ando K. Enhanced aldosterone signaling in the early nephropathy of rats with metabolic syndrome: possible contribution of fat-derived factors. J Am Soc Nephrol. (2006) 17:3438–46. doi: 10.1681/ASN.2006080944 17082236

[44] Packer M. Leptin-aldosterone-neprilysin axis: identification of its distinctive role in the pathogenesis of the three phenotypes of heart failure in people with obesity. Circulation. (2018) 137:1614–31. doi: 10.1161/CIRCULATIONAHA.117.032474 29632154

[45] Cohen J Bellomo R Billot L Burrell LM Evans DM Finfer S. Plasma cortisol, aldosterone, and ascorbic acid concentrations in patients with septic shock do not predict treatment effect of hydrocortisone on mortality. A nested cohort study. Am J Respir Crit Care Med. (2020) 202:700–7. doi: 10.1164/rccm.202002-0281OC 32396775

[46] Fallo F Dalla Pozza A Tecchio M Tona F Sonino N Ermani M. Nonalcoholic fatty liver disease in primary aldosteronism: a pilot study. Am J Hypertens. (2010) 23:2–5. doi: 10.1038/ajh.2009.206 19910932

[47] Zhang X Wong GL Yip TC Tse YK Liang LY Hui VW. Angiotensin-converting enzyme inhibitors prevent liver-related events in nonalcoholic fatty liver disease. Hepatology. (2022) 76:469–82. doi: 10.1002/hep.32294 34939204

[48] Polyzos SA Kountouras J Zafeiriadou E Patsiaoura K Katsiki E Deretzi G. Effect of spironolactone and vitamin E on serum metabolic parameters and insulin resistance in patients with nonalcoholic fatty liver disease. J Renin Angiotensin Aldosterone Syst. (2011) 12:498–503. doi: 10.1177/1470320311402110 21436212

[49] Kumar A Blackshear C Subauste JS Esfandiari NH Oral EA Subauste AR. Fatty liver disease, women, and aldosterone: finding a link in the jackson heart study. J Endocr Soc. (2017) 1:460–9. doi: 10.1210/js.2017-00055 PMC5686785 29264501

[50] Srinivasa S Fitch KV Quadri N Maehler P O'Malley TK Martinez-Salazar EL. Significant association of aldosterone and liver fat among HIV-infected individuals with metabolic dysregulation. J Endocr Soc. (2018) 2:1147–57. doi: 10.1210/js.2018-00194 PMC6162603 30283827

[51] Huby T Gautier EL. Immune cell-mediated features of non-alcoholic steatohepatitis. Nat Rev Immunol. (2022) 22:429–43. doi: 10.1038/s41577-021-00639-3 PMC8570243 34741169

[52] Wu X Azizan E Goodchild E Garg S Hagiyama M Cabrera CP. Somatic mutations of CADM1 in aldosterone-producing adenomas and gap junction-dependent regulation of aldosterone production. Nat Genet. (2023) 55:1009–21. doi: 10.1038/s41588-023-01403-0 PMC10260400 37291193

[53] Spät A Hunyady L. Control of aldosterone secretion: a model for convergence in cellular signaling pathways. Physiol Rev. (2004) 84:489–539. doi: 10.1152/physrev.00030.2003 15044681

[54] Yuan M He J Hu X Yao L Chen P Wang Z. Hypertension and NAFLD risk: Insights from the NHANES 2017-2018 and Mendelian randomization analyses. Chin Med J (Engl). (2024) 137:457–64. doi: 10.1097/CM9.0000000000002753 PMC10876227 37455323

[55] Villanueva MT. Organoids illuminate NAFLD pathogenesis. Nat Rev Drug Discov. (2023) 22:269. doi: 10.1038/d41573-023-00047-3 36899272

[56] White PC Mune T Agarwal AK. 11 beta-Hydroxysteroid dehydrogenase and the syndrome of apparent mineralocorticoid excess. Endocr Rev. (1997) 18:135–56. doi: 10.1210/edrv.18.1.0288 9034789

[57] Rossier BC Baker ME Studer RA. Epithelial sodium transport and its control by aldosterone: the story of our internal environment revisited. Physiol Rev. (2015) 95:297–340. doi: 10.1152/physrev.00011.2014 25540145

[58] Monteiro S Grandt J Uschner FE Kimer N Madsen JL Schierwagen R. Differential inflammasome activation predisposes to acute-on-chronic liver failure in human and experimental cirrhosis with and without previous decompensation. Gut. (2021) 70:379–87. doi: 10.1136/gutjnl-2019-320170 PMC7815638 32241903

[59] Liu S Liu Y Zhang D Li H Shao X Xie P. Novel insights into perfluorinated compound-induced hepatotoxicity: Chronic dietary restriction exacerbates the effects of PFBS on hepatic lipid metabolism in mice. Environ Int. (2023) 181:108274. doi: 10.1016/j.envint.2023.108274 37879206

[60] Harrison SA Bedossa P Guy CD Schattenberg JM Loomba R Taub R. A phase 3, randomized, controlled trial of resmetirom in NASH with liver fibrosis. N Engl J Med. (2024) 390:497–509. doi: 10.1056/NEJMoa2309000 38324483

[61] Boyer-Diaz Z Aristu-Zabalza P Andrés-Rozas M Robert C Ortega-Ribera M Fernández-Iglesias A. Pan-PPAR agonist lanifibranor improves portal hypertension and hepatic fibrosis in experimental advanced chronic liver disease. J Hepatol. (2021) 74:1188–99. doi: 10.1016/j.jhep.2020.11.045 33278455

