# Supplementary material for: Associating plasma aldosterone concentration with the prevalence of MAFLD in hypertensive patients: insights from a large-scale cross-sectional study
Source: Front Endocrinol (Lausanne). 2024 Sep 19;15:1451383. doi: 10.3389/fendo.2024.1451383 (PMC11446807; doi:10.3389/fendo.2024.1451383)
Supplement: Supplementary file 1 [file DataSheet1.docx]

Supplementary Material

# Supplemental material and methods

- 1. **Baseline examination**

Anthropometric measurements were taken by trained nurses. Data for height and weight were 3 acquired following a protocol standardized to an accuracy of 0.1 kg and 0.1 cm, respectively. The waist circumference (WC) is measured at the midpoint between the lower margin of the last palpable rib and the top of the iliac crest in a horizontal plane, with the individual standing and feet together. The measurement is taken at the end of a normal expiration, and two measurements are recorded for accuracy, with the average of the closest two measurements used. Current smokers were defined as having smoked 100 cigarettes in their lifetime and currently smoking. Alcohol consumption was evaluated with questions regarding the types of alcoholic beverages, the frequency of alcohol consumption per week, and the usual amount consumed per occasion. Subjects who reported alcohol consumption >140 g/week for men and >70 g/week for women were deemed to have excessive alcohol consumption ^[1]^. Blood pressure was measured using a mercury sphygmomanometer after the patient had rested quietly for at least 10 minutes, and the average of multiple measurements was taken as the systolic and diastolic blood pressure values. All biochemical tests were measured by blood sampling after an overnight fast. Plasma aldosterone concentrations (PAC) was measured using radio-immunoassay (DSL-8600 ACTIVE Aldosterone Coated Tube Radioimmunoassay Kit; Diagnostic Systems Laboratories, Webster, TX). For the measurement of PAC, participants were asked to sit for 30 minutes after being active for at least 2 hours before blood collection, which took place between 8:00 and 11:00 AM.

**1.2 Definition**

The body mass index (BMI) was calculated as weight divided by height squared. Excessive drinking can be defined as consuming alcohol >20 g/day for women or >30 g/day for men. Type 2 diabetes mellitus (T2DM) was defined as fasting serum glucose ≥7.0 mmol/L, the 2-h serum glucose of the oral glucose tolerance test ≥11.1 mmol/L, or the current use of hypoglycaemic medication or insulin. Dyslipidemia was defined by self-report dyslipidemia and/or treatment with a lipid-lowering drug. The diagnosis of coronary artery disease (CAD) was based on the patient's self-reported history of myocardial infarction, percutaneous coronary intervention, or coronary bypass grafting. Criteria for hypertension included self-reported hypertension, current use of anti-hypertensive medication, or systolic blood pressure (SBP) ≥ 140 mmHg and/or diastolic blood pressure (DBP) ≥ 90 mmHg recorded for at least three consecutive readings. A patient was diagnosed with PA as per the Endocrine Society’s clinical practice guidelines, based on PAC levels ≥ 12 ng/dL, aldosterone-to-renin ratio ≥ 20, and a PAC value ≥ 10 ng/dL confirmed by saline infusion test ^[2]^. Advanced fibrosis was defined as NFS ≥0.676 or FIB-4 score > 2.67 at baseline. Advanced fibrosis was excluded if there was a NFS < −1.455 (< 0.12 if ≥ 65 years) or FIB-4 score < 1.3 (< 2.0 if ≥ 65 years) ^[3]^. We selected NFS and FIB-4 to identify participants with liver fibrosis because they are superior to other scores that predict the presence of liver fibrosis ^[4]^.

**1.3 Details of the statistical analyses.**

Clinical and demographic characteristics were reported using proportions, the mean and standard deviation (SD), or the median and interquartile range (IQR), as appropriate. To compare the characteristics among different PAC groups, the chi-square test was performed for categorical variables, and one-way analysis of variance, or the Kruskal-Wallis test, was performed for continuous variables with normal and skewed distributions. We used nonparametric missing value imputation, based on the missForest procedure in R, to account for missing data. A random forest model using the rest of the variables in the data set was performed to predict the missing values for these variables. The internally cross-validated errors were also estimated.

Multicollinearity in the resulting models was quantified using the variance inflation factor (VIF)—variables with VIF ≥ 5 were removed (Table S1). Then multivariable logistic regression models [odds ratio (OR) and 95% confidence interval (CI)] were used to evaluate the relationship between PAC and prevalence of MAFLD. Model 1 did not adjust for covariates; model 2 adjusted for age, sex, smoking status, alcohol consumption, BMI, SBP, and DBP; model 3 adjusted for variables in Model 2+ diabetes mellitus, hyperlipidemia, CAD; model 4 adjusted for variables in Model 3+ Serum potassium, Serum sodium, PLT, ALT, AST, GGT, Cr, BUN, UA, Total cholesterol, Triglyceride, HDL-C, LDL-C, FBG, HbA1c, hs-CRP, triglyceride–glucose (TyG) index; and model 5 adjusted for variables in Model 4+ angiotensin converting enzyme inhibitors (ACEI) / angiotensin II receptor blockers (ARB), diuretic, calcium channel blockers (CCB), β-blockers, antidiabetic agents, lipid-lowering drugs. P value for trend was calculated by treating quartiles as a continuous variable in each model. We used restricted cubic spline (RCS) models fitted for linear regression models and logistic regression models. Knots were present at the 5th, 25th, 75th, and 95th percentiles. The RCS, based on the linear regression model, was used to model the association between PAC and prevalence of MAFLD.

As additional exploratory analyses, possible modifications of the association between PAC and prevalence of MAFLD were also assessed for variables including sex, age (years; <60 compared with ≥60), BMI (in kg/m^2^; <24 compared with ≥24), current smoking (yes or no), current drinking (yes or no), diabetes mellitus (yes or no), hyperlipidemia (yes or no), and CAD (yes or no). Then, conduct subgroup analysis based on the use of different medications (ACEI/ARB, diuretic, CCB, beta-blockers, antidiabetic agents, lipid-lowering drugs). The statistical significance of an interaction term was evaluated by using the likelihood ratio test. We did sensitivity analyses. First, to assess the impact of missing data, we repeated the preliminary analysis after excluding subjects with missing covariates at baseline. Second, in order to evaluate the impact of alcohol abuse, we repeated the main analysis after excluding participants with alcohol abuse at baseline. Furthermore, to evaluate the impact of severe liver fibrosis, we repeated the primary analysis after excluding participants with severe liver fibrosis at baseline.

The statistical analysis was performed using software R, version 4.1.1 (R Foundation for Statistical Computing), and significance was set at a 2-sided P value less than 0.05.

# Supplementary Figures and Tables

## Supplementary Tables

**Table S1.** Collinearity diagnostics steps.

|  | Step 1 | Step 2 |
| --- | --- | --- |
| PAC | 4.3 | 4.3 |
| Age | 1.3 | 1.3 |
| Sex | 1.8 | 1.8 |
| BMI | 1.2 | 1.2 |
| WC | 1 | 1 |
| SBP | 1.8 | 1.8 |
| DBP | 1.9 | 1.9 |
| Current smoking | 1.8 | 1.8 |
| Current drinking | 1.7 | 1.7 |
| PLT | 1.1 | 1.1 |
| ALT | 6.9 | 6.9 |
| AST | 4.9 | 4.9 |
| GGT | 1.6 | 1.6 |
| Scr | 1.6 | 1.5 |
| BUN | 1.2 | 1.2 |
| UA | 1.6 | 1.6 |
| Total cholesterol | 1.6 | 1.6 |
| Triglyceride | 1.8 | 1.8 |
| HDL-C | 1.7 | 1.7 |
| LDL-C | 1.1 | 1.1 |
| FBG | 2.4 | 2.4 |
| HbA1c | 2.6 | 2.6 |
| hs-CRP | 1 | 1 |
| TyG index | 4.3 | 4.3 |
| T2DM | 2.7 | 2.7 |
| Dyslipidemia | 1.1 | 1.1 |
| CAD | 1.2 | 1.2 |
| ACEI/ARB | 1.2 | 1.2 |
| Diuretic | 1.2 | 1.2 |
| CCB | 1.1 | 1.1 |
| β-blockers | 1.1 | 1.1 |
| Antidiabetic agents | 1.6 | 1.6 |
| Lipid-lowering drugs | 1.2 | 1.2 |

VIF = 1/(1-R^2^). VIF step-by-step screening method: Calculate the VIF of each variable. If the maximum VIF value ≥ 5, remove the variable with the maximum VIF value.

Abbreviations: VIF, variance inflation factor. Other abbreviations as presented in Table 1.

**Table S2.** Baseline characteristics of participants with or without MAFLD.

| MAFLD | No | Yes | P-value |
| --- | --- | --- | --- |
| Sample size, n | 16721 | 18438 |  |
| PAC, ng/dL | 14.33 ± 4.90 | 16.88 ± 5.78 | <0.001 |
| Demography |  |  |  |
| Age, years | 51.10 ± 12.12 | 50.66 ± 12.05 | <0.001 |
| Sex, % |  |  | 0.421 |
| Women | 7135 (42.67%) | 7946 (43.10%) |  |
| Men | 9586 (57.33%) | 10492 (56.90%) |  |
| BMI, kg/m^2^ | 26.91 ± 3.63 | 26.95 ± 3.64 | 0.259 |
| WC, cm | 95.89 ± 11.28 | 95.82 ± 11.18 | 0.568 |
| SBP, mmHg | 145.98 ± 18.35 | 146.05 ± 18.24 | 0.732 |
| DBP, mmHg | 88.02 ± 13.56 | 88.19 ± 13.63 | 0.233 |
| Current smoking, % | 5723 (34.23%) | 5989 (32.48%) | <0.001 |
| Current drinking, % | 5223 (31.24%) | 5625 (30.51%) | 0.140 |
| Biochemical indexes |  |  |  |
| PLT, 10^9/L | 242.63 ± 58.40 | 241.60 ± 57.81 | 0.095 |
| ALT, U/L | 27.26 ± 17.54 | 27.30 ± 17.51 | 0.842 |
| AST, U/L | 21.09 ± 8.24 | 21.03 ± 8.21 | 0.500 |
| GGT, U/L | 35.72 ± 25.12 | 36.12 ± 25.39 | 0.132 |
| Scr, µmol/L | 65.05 ± 14.36 | 65.16 ± 14.45 | 0.449 |
| BUN, mmol/L | 5.05 ± 1.36 | 5.06 ± 1.36 | 0.180 |
| UA, umol/L | 343.40 ± 91.05 | 344.30 ± 91.02 | 0.351 |
| Total cholesterol, mmol/L | 4.53 ± 0.98 | 4.54 ± 0.97 | 0.419 |
| Triglyceride, mmol/L | 1.81 ± 1.04 | 1.82 ± 1.03 | 0.298 |
| HDL-C, mmol/L | 1.06 ± 0.25 | 1.06 ± 0.25 | 0.151 |
| LDL-C, mmol/L | 2.76 ± 0.82 | 2.75 ± 0.83 | 0.840 |
| FBG, mmol/L | 5.02 ± 1.04 | 5.03 ± 1.05 | 0.546 |
| HbA1c, % | 5.94 ± 0.79 | 5.93 ± 0.79 | 0.455 |
| hs-CRP, mg/dL | 3.49 ± 3.06 | 3.53 ± 3.09 | 0.212 |
| TyG index | 7.02 ± 0.60 | 7.30 ± 0.66 | <0.001 |
| T2DM, % | 2663 (15.93%) | 2975 (16.14%) | 0.594 |
| Dyslipidemia, % | 3240 (19.38%) | 3425 (18.58%) | 0.056 |
| CAD, % | 1563 (9.35%) | 1680 (9.11%) | 0.445 |
| Medications use |  |  |  |
| ACEI/ARB, % | 7728 (46.22%) | 8499 (46.10%) | 0.818 |
| Diuretic, % | 1793 (10.72%) | 1994 (10.81%) | 0.782 |
| CCB, % | 4254 (25.44%) | 4729 (25.65%) | 0.657 |
| β-blockers, % | 3016 (18.04%) | 3208 (17.40%) | 0.117 |
| Antidiabetic agents, % | 1243 (7.43%) | 1373 (7.45%) | 0.964 |
| Lipid-lowering drugs, % | 1948 (11.65%) | 2149 (11.66%) | 0.988 |

Abbreviations: See Table 1.

**Table S3**. Sensitivity analysis excludes participants with any missing values.

| Exposure | Model 1  OR (95% CI) | Model 2  OR (95% CI) | Model 3  OR (95% CI) | Model 4  OR (95% CI) | Model 5  OR (95% CI) |
| --- | --- | --- | --- | --- | --- |
| PAC (per 5-SD increase) | 1.57 (1.53, 1.61) | 1.57 (1.53, 1.60) | 1.57 (1.53, 1.60) | 1.56 (1.52, 1.59) | 1.56 (1.52, 1.60) |
| PAC quartiles |  |  |  |  |  |
| Q1 | Reference | Reference | Reference | Reference | Reference |
| Q2 | 1.18 (1.11, 1.27) | 1.18 (1.11, 1.26) | 1.18 (1.11, 1.26) | 1.18 (1.10, 1.26) | 1.18 (1.10, 1.26) |
| Q3 | 2.14 (2.00, 2.28) | 2.13 (1.99, 2.28) | 2.13 (1.99, 2.28) | 2.12 (1.98, 2.26) | 2.12 (1.98, 2.27) |
| Q4 | 3.15 (2.94, 3.37) | 3.13 (2.92, 3.35) | 3.12 (2.92, 3.35) | 3.06 (2.86, 3.29) | 3.09 (2.88, 3.31) |
| P for trend | <0.001 | <0.001 | <0.001 | <0.001 | <0.001 |

Model 1: crude model.

Model 2: adjusted for age, sex, smoking status, alcohol consumption, BMI, SBP, and DBP.

Model 3: adjusted for variables in Model 2 plus T2DM, dyslipidemia, CAD.

Model 4: adjusted for variables in Model 3 plus Serum potassium, Serum sodium, PLT, ALT, AST, GGT, Scr, BUN, UA, Total cholesterol, Triglyceride, HDL-C, LDL-C, FBG, HbA1c, hs-CRP, TyG index.

Model 5: adjusted for variables in Model 4 plus ACEI/ARB, diuretic, CCB, β-blockers, antidiabetic agents, lipid-lowering drugs.

Abbreviations: SD, standard deviation; OR, odds ratio; CI, confidence interval. Other abbreviations, see Table 1.

**Table S4.** Sensitivity analysis excludes participants with alcohol abuse.

| Exposure | Model 1  OR (95% CI) | Model 2  OR (95% CI) | Model 3  OR (95% CI) | Model 4  OR (95% CI) | Model 5  OR (95% CI) |
| --- | --- | --- | --- | --- | --- |
| PAC (per 5-SD increase) | 1.55 (1.51, 1.59) | 1.55 (1.51, 1.59) | 1.55 (1.51, 1.59) | 1.54 (1.50, 1.58) | 1.54 (1.50, 1.58) |
| PAC quartiles |  |  |  |  |  |
| Q1 | Reference | Reference | Reference | Reference | Reference |
| Q2 | 1.23 (1.15, 1.33) | 1.23 (1.15, 1.32) | 1.23 (1.15, 1.33) | 1.23 (1.14, 1.32) | 1.23 (1.14, 1.32) |
| Q3 | 2.13 (1.98, 2.29) | 2.12 (1.98, 2.28) | 2.13 (1.98, 2.29) | 2.11 (1.97, 2.27) | 2.12 (1.97, 2.28) |
| Q4 | 3.11 (2.88, 3.34) | 3.08 (2.86, 3.32) | 3.09 (2.87, 3.33) | 3.02 (2.80, 3.25) | 3.03 (2.81, 3.27) |
| P for trend | <0.001 | <0.001 | <0.001 | <0.001 | <0.001 |

Model 1: crude model.

Model 2: adjusted for age, sex, smoking status, alcohol consumption, BMI, SBP, and DBP.

Model 3: adjusted for variables in Model 2 plus T2DM, dyslipidemia, CAD.

Model 4: adjusted for variables in Model 3 plus Serum potassium, Serum sodium, PLT, ALT, AST, GGT, Scr, BUN, UA, Total cholesterol, Triglyceride, HDL-C, LDL-C, FBG, HbA1c, hs-CRP, TyG index.

Model 5: adjusted for variables in Model 4 plus ACEI/ARB, diuretic, CCB, β-blockers, antidiabetic agents, lipid-lowering drugs.

Abbreviations: SD, standard deviation; OR, odds ratio; CI, confidence interval. Other abbreviations, see Table 1.

**Table S5.** Sensitivity analysis excludes participants with severe liver fibrosis (NFS ≥ 0.676).

| Exposure | Model 1  OR (95% CI) | Model 2  OR (95% CI) | Model 3  OR (95% CI) | Model 4  OR (95% CI) | Model 5  OR (95% CI) |
| --- | --- | --- | --- | --- | --- |
| PAC (per 5-SD increase) | 1.57 (1.54, 1.61) | 1.57 (1.54, 1.60) | 1.57 (1.54, 1.60) | 1.56 (1.53, 1.59) | 1.56 (1.53, 1.60) |
| PAC quartiles |  |  |  |  |  |
| Q1 | Reference | Reference | Reference | Reference | Reference |
| Q2 | 1.21 (1.14, 1.29) | 1.21 (1.14, 1.29) | 1.21 (1.14, 1.29) | 1.21 (1.14, 1.28) | 1.21 (1.14, 1.28) |
| Q3 | 2.12 (2.00, 2.25) | 2.12 (1.99, 2.25) | 2.12 (2.00, 2.25) | 2.11 (1.98, 2.24) | 2.11 (1.99, 2.25) |
| Q4 | 3.20 (3.01, 3.41) | 3.19 (3.00, 3.40) | 3.19 (3.00, 3.40) | 3.13 (2.94, 3.33) | 3.15 (2.95, 3.35) |
| P for trend | <0.001 | <0.001 | <0.001 | <0.001 | <0.001 |

Model 1: crude model.

Model 2: adjusted for age, sex, smoking status, alcohol consumption, BMI, SBP, and DBP.

Model 3: adjusted for variables in Model 2 plus T2DM, dyslipidemia, CAD.

Model 4: adjusted for variables in Model 3 plus Serum potassium, Serum sodium, PLT, ALT, AST, GGT, Scr, BUN, UA, Total cholesterol, Triglyceride, HDL-C, LDL-C, FBG, HbA1c, hs-CRP, TyG index.

Model 5: adjusted for variables in Model 4 plus ACEI/ARB, diuretic, CCB, β-blockers, antidiabetic agents, lipid-lowering drugs.

Abbreviations: SD, standard deviation; OR, odds ratio; CI, confidence interval; NFS=Non-alcoholic Fatty Liver Disease Fibrosis Score. Other abbreviations, see Table 1.

**Table S6.** Sensitivity analysis excludes participants with severe liver fibrosis (FIB-4 > 2.67).

| Exposure | Model 1  OR (95% CI) | Model 2  OR (95% CI) | Model 3  OR (95% CI) | Model 4  OR (95% CI) | Model 5  OR (95% CI) |
| --- | --- | --- | --- | --- | --- |
| PAC (per 5-SD increase) | 1.57 (1.54, 1.60) | 1.57 (1.54, 1.60) | 1.57 (1.54, 1.60) | 1.56 (1.53, 1.59) | 1.56 (1.53, 1.60) |
| PAC quartiles |  |  |  |  |  |
| Q1 | Reference | Reference | Reference | Reference | Reference |
| Q2 | 1.21 (1.14, 1.29) | 1.21 (1.14, 1.29) | 1.21 (1.14, 1.29) | 1.21 (1.14, 1.28) | 1.21 (1.14, 1.28) |
| Q3 | 2.12 (2.00, 2.26) | 2.12 (2.00, 2.25) | 2.12 (2.00, 2.26) | 2.11 (1.99, 2.24) | 2.12 (1.99, 2.25) |
| Q4 | 3.19 (3.00, 3.39) | 3.18 (2.99, 3.38) | 3.18 (2.99, 3.38) | 3.12 (2.93, 3.32) | 3.13 (2.94, 3.34) |
| P for trend | <0.001 | <0.001 | <0.001 | <0.001 | <0.001 |

Model 1: crude model.

Model 2: adjusted for age, sex, smoking status, alcohol consumption, BMI, SBP, and DBP.

Model 3: adjusted for variables in Model 2 plus T2DM, dyslipidemia, CAD.

Model 4: adjusted for variables in Model 3 plus Serum potassium, Serum sodium, PLT, ALT, AST, GGT, Scr, BUN, UA, Total cholesterol, Triglyceride, HDL-C, LDL-C, FBG, HbA1c, hs-CRP, TyG index.

Model 5: adjusted for variables in Model 4 plus ACEI/ARB, diuretic, CCB, β-blockers, antidiabetic agents, lipid-lowering drugs.

Abbreviations: SD, standard deviation; OR, odds ratio; CI, confidence interval; FIB-4, Fibrosis-4 score, Other abbreviations, see Table 1.

**Table S7**. E-values for the observed associations between PAC and MAFLD.

|  | Model 1 | Model 2 | Model 3 | Model 4 | Model 5 |
| --- | --- | --- | --- | --- | --- |
| OR (95% CI) (per 5 ng/dl increase) | 1.57 (1.54, 1.61) | 1.57 (1.54, 1.60) | 1.57 (1.54, 1.60) | 1.56 (1.53, 1.60) | 1.56 (1.53, 1.60) |
| E-value for point estimate | 2.52 | 2.52 | 2.52 | 2.49 | 2.49 |

Model 1: crude model.

Model 2: adjusted for age, sex, smoking status, alcohol consumption, BMI, SBP, and DBP.

Model 3: adjusted for variables in Model 2 plus T2DM, dyslipidemia, CAD.

Model 4: adjusted for variables in Model 3 plus Serum potassium, Serum sodium, PLT, ALT, AST, GGT, Scr, BUN, UA, Total cholesterol, Triglyceride, HDL-C, LDL-C, FBG, HbA1c, hs-CRP, TyG index.

Model 5: adjusted for variables in Model 4 plus ACEI/ARB, diuretic, CCB, β-blockers, antidiabetic agents, lipid-lowering drugs.

Abbreviations: SD, standard deviation; OR, odds ratio; CI, confidence interval. Other abbreviations, see Table 1.

## 2.2 Supplementary Figure


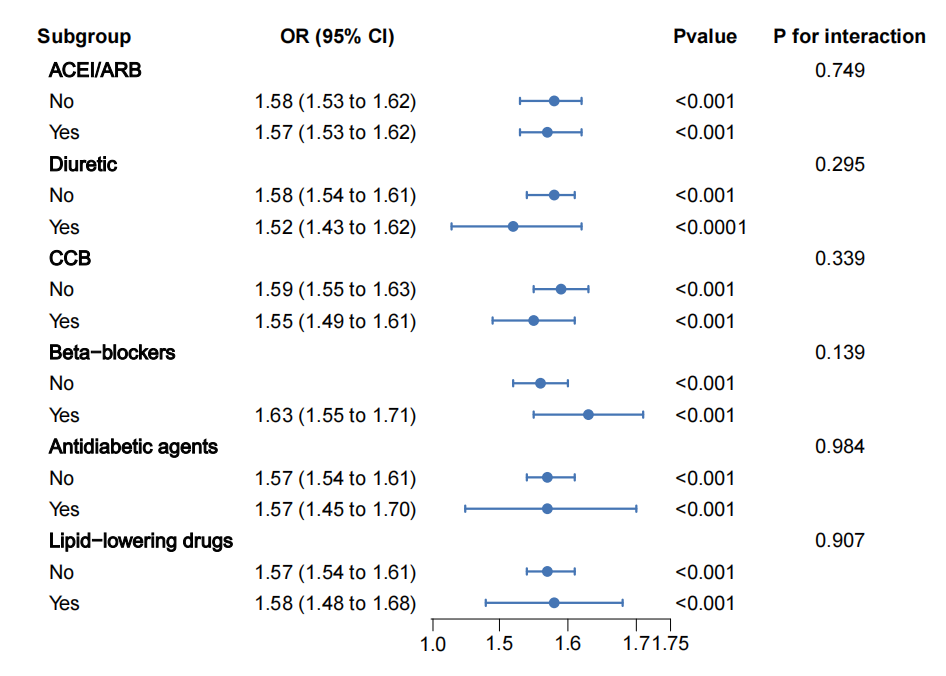


**Figure S1** Stratified analyses of the association between PAC (per 5 ng/dL increment) and the prevalence of MAFLD in hypertensive patients.


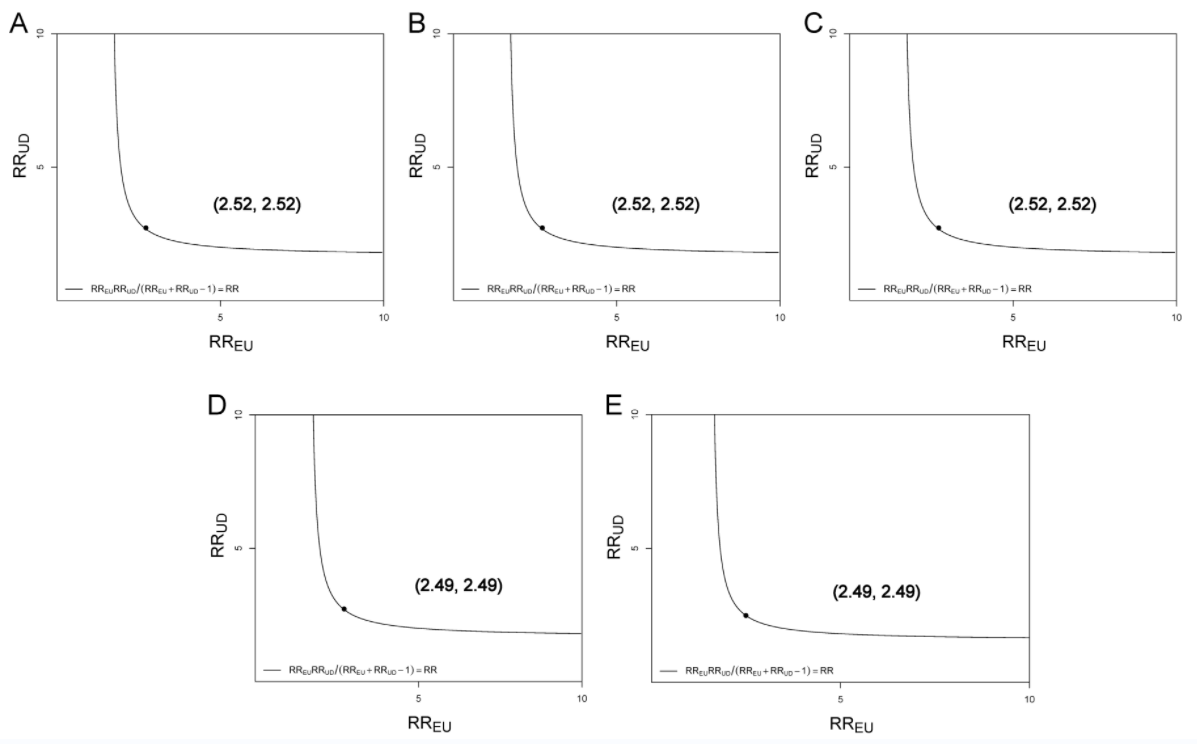


**Figure S2** E-values for multivariable logistic regression models.

**References**

[1] Farrell GC, Chitturi S, Lau GK, Sollano JD. Guidelines for the assessment and management of non-alcoholic fatty liver disease in the Asia-Pacific region: executive summary. J Gastroenterol Hepatol. 2007. 22(6): 775-7.

[2] Funder JW, Carey RM, Mantero F, et al. The Management of Primary Aldosteronism: Case Detection, Diagnosis, and Treatment: An Endocrine Society Clinical Practice Guideline. J Clin Endocrinol Metab. 2016. 101(5): 1889-916.

[3] Paik J, Golabi P, Younoszai Z, Mishra A, Trimble G, Younossi ZM. Chronic kidney disease is independently associated with increased mortality in patients with nonalcoholic fatty liver disease. Liver Int. 2019. 39(2): 342-352.

[4] Shah AG, Lydecker A, Murray K, Tetri BN, Contos MJ, Sanyal AJ. Comparison of noninvasive markers of fibrosis in patients with nonalcoholic fatty liver disease. Clin Gastroenterol Hepatol. 2009. 7(10): 1104-12.
